# Functional Relationship between Tumor-Associated Macrophages and Macrophage Colony-Stimulating Factor as Contributors to Cancer Progression

**DOI:** 10.3389/fimmu.2014.00489

**Published:** 2014-10-07

**Authors:** Damya Laoui, Eva Van Overmeire, Patrick De Baetselier, Jo A. Van Ginderachter, Geert Raes

**Affiliations:** ^1^Myeloid Cell Immunology Laboratory, VIB, Brussels, Belgium; ^2^Unit of Cellular and Molecular Immunology, Vrije Universiteit Brussel, Brussels, Belgium

**Keywords:** M-CSF, CSF-1, M-CSFR, CSF-1R, tumor-associated macrophages, M1, M2, cancer progression

## Abstract

The current review article describes the functional relationship between tumor-associated macrophages (TAM) as key cellular contributors to cancer malignancy on the one hand and macrophage-colony-stimulating factor (M-CSF or CSF-1) as an important molecular contributor on the other. We recapitulate the available data on expression of M-CSF and the M-CSF receptor (M-CSFR) in human tumor tissue as constituents of a stromal macrophage signature and on the limits of the predictive and prognostic value of plasma M-CSF levels. After providing an update on current insights into the nature of TAM heterogeneity at the level of M1/M2 phenotype and TAM subsets, we give an overview of experimental evidence, based on genetic, antibody-mediated, and pharmacological disruption of M-CSF/M-CSFR signaling, for the extent to which M-CSFR signaling can not only determine the TAM quantity, but can also contribute to shaping the phenotype and heterogeneity of TAM and other related tumor-infiltrating myeloid cells (TIM). Finally, we review the accumulating information on the – sometimes conflicting – effects blocking M-CSFR signaling may have on various aspects of cancer progression such as tumor growth, invasion, angiogenesis, metastasis, and resistance to therapy and we thereby discuss in how far these different effects actually reflect a contribution of TAM.

## Introduction

### Cancer malignancy

Ca ncer is a complex multi-step process, in which normal cells acquire a certain growth advantage via a process analogous to Darwinian evolution. These cellular changes can occur under many different circumstances, which contributes to the heterogeneity and variability of the occurrence, development, and outcome of neoplastic disease (1). The traits required for malignant growth include self-sufficiency from external growth signals, insensitivity to negative growth signals, resistance to apoptosis, limitless replicative potential, sustained angiogenesis, acquisition of tissue invasiveness, and metastasis. Recently, genetic instability, altered energy metabolism, the capacity to evade elimination by the immune system, including active immune suppression, as well as smoldering, non-resolving inflammation, leading to accumulation of random genetic alterations in cancer cells due to inflammatory mediators, have been established as additional hallmarks of cancer (2–6). In this regard, tumors consist not only of neoplastic cells, but should be considered as organ-like structures in which a complex bidirectional interplay exists between transformed and non-transformed cells. The malignant potential of transformed cells requires an apt support structure from the stroma, which can consist of fibroblasts, adipocytes, blood, and lymph vessels, but may also be considerably infiltrated by a wide range of immune cells, such as tumor-associated macrophages (TAM) (7).

### Pro- and anti-tumoral roles of TAM

Tumor-associated macrophages are the predominant leukocytes infiltrating solid tumors and can represent up to 50% of the tumor mass. The clinical significance of these cells is illustrated by the significant link between TAM number and density and a poor prognosis in 80% of the reported studies. The main exception to this general trend seems to be colorectal cancer, for which a high TAM density is significantly associated with enhanced overall survival (8–10).

Tumor-associated macrophages stimulated by TLR ligands, agonistic anti-CD40, or IFN-γ were shown to have important anti-tumoral activities, provided that cancer cell phagocytosis is not inhibited by CD47 expression on the malignant cells, which is a “don’t-eat me signal” (11, 12). In addition, pro-inflammatory macrophages are able to eliminate cancer cells via the production of reactive oxygen species (ROS) and reactive nitrogen intermediates (RNI) and secrete chemokines that recruit and prime T cells toward an anti-tumor phenotype in some cancer types, resulting in retarded tumor growth or tumor regression (13–17).

**Figure 1:**
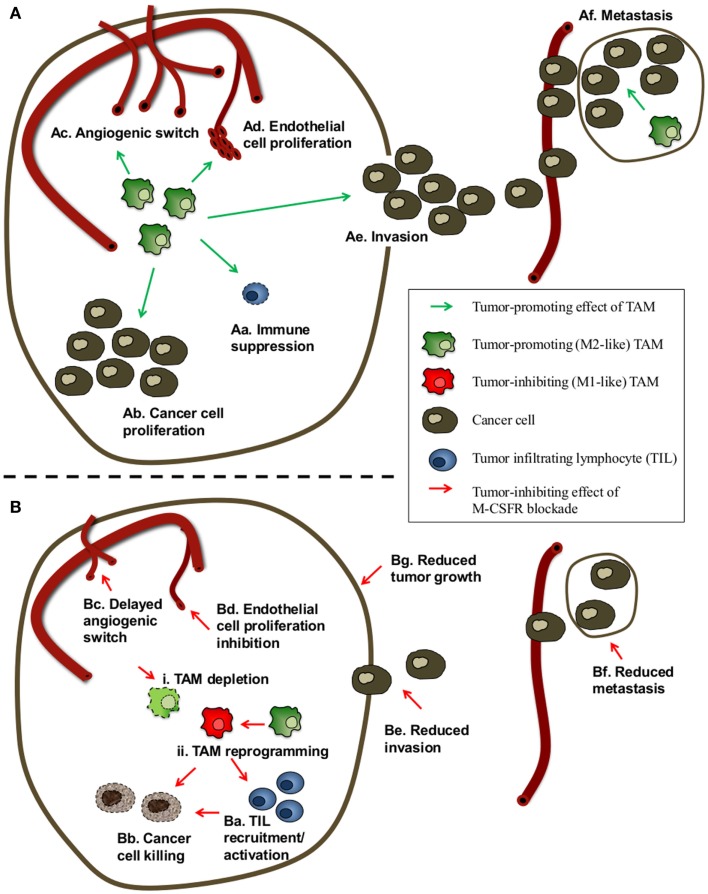
**Scheme of the possible effects of TAM and of M-CSFR blockade on cancer progression**. **(A)** Possible tumor-promoting effects of TAM. TAM can promote cancer progression and reduce the efficacy of radiotherapy, chemotherapy, immunotherapy, and anti-angiogenic therapy by a combination of different mechanisms. TAM can contribute to enhanced cancer cell numbers by (Aa) inhibiting anti-tumor immune responses and via (Ab) stimulation/maintenance of cancer cell proliferation. TAM can also exert pro-angiogenic activities by enhancing (Ac) angiogenic switching and (Ad) endothelial cell proliferation. Finally, TAM can contribute to cancer malignancy by facilitating (Ae) cancer cell invasion and (Af) seeding, extravasation, survival, and subsequent proliferation of cancer cells at metastatic sites. **(B)** Possible effects of M-CSFR signaling blockade on cancer progression. Depending on the tumor type/model and the blocking agents used to impede M-CSFR signaling, M-CSFR blockade has in most cases been reported to attenuate cancer progression and/or synergistically enhance the effect of chemo-, radio-, and/or immunotherapy via various effects, including (Ba) promotion of tumor-infiltrating lymphocytes (TIL) recruitment and/or activation, (Bb) enhanced phagocytosis/killing of cancer cells, (Bc) a delayed angiogenic switch, (Bd) reduced density of proliferating endothelial cells, (Be) inhibition of both TAM and cancer cell migration and invasion, (Bf) reduced metastasis. In some cases, (Bg) reduction of tumor weight and primary tumor growth has been reported. A number of studies have attributed these effects to (i) ablation of TAM numbers and/or (ii) phenotypic reprograming of TAM from tumor promoting (often M2-like) TAM to anti-tumor (often M1-like) TAM.

Whereas TAM can exert anti-tumoral activities, the ambiguous role of macrophages in tumor progression is reflected in the finding that TAM can also actively contribute to each stage of cancer development and progression (Figure 1A). They can promote cancer cell proliferation, invasion, metastasis, and angiogenesis by releasing cytokines, growth factors, extracellular matrix-degrading enzymes, and angiogenic factors including vascular endothelial growth factor (VEGF), prokineticin (Bv8), and matrix metalloproteinase 9 (MMP9). TAM also inhibit cytotoxic T-cell activity by the secretion of suppressive cytokines such as IL-10 and transforming growth factor-β (TGF-β), high levels of arginase activity, and the production of ROS or RNI (18–22). Finally, TAM contribute to tumor relapse following tumor irradiation and anti-angiogenic therapy (23).

It seems unlikely that the diverse anti-tumoral and tumor-promoting activities of TAM are performed by a single cell type, and the existence of distinct TAM subpopulations, linked to different intratumoral microenvironments, has been predicted (10, 24). Depending on the cancer type, the stage of tumor progression and location within the tumor tissue, molecularly and functionally distinct TAM subpopulations coexist in tumors (25–27). This TAM heterogeneity likely reflects the inherent plasticity of macrophages in response to (micro-)environmental triggers.

### Macrophage plasticity

Macrophages have a remarkable plasticity and are found in all tissues, where they display great anatomical and functional diversity. They are implicated in a spectrum of roles required for tissue homeostasis, ranging from host defense against infectious agents, to tissue development, wound healing, and immune regulation. Accordingly, macrophages are able to adopt diverse phenotypes or activation states in response to environmental cues, such as cytokines, pathogen-associated molecular patterns (PAMP), tissue damage-associated molecular patterns (DAMP), and other immune stimuli (7, 28, 29).

Macrophage activation is conventionally categorized on a linear scale, in which the two opposing phenotypes are referred to as the classical (M1) versus alternative (M2) macrophage activation state, originally mirroring the Th1 versus Th2 nomenclature (30–34). M1 macrophage activation is driven by exposure to IFN-γ and TLR ligands. These macrophages secrete pro-inflammatory cytokines (such as IL-12, IL-1, IL-6, TNF, ROS, RNI), promote Th1 responses, exert cytotoxic activities, and are involved in defense against bacterial infections and intracellular pathogens. The M2 activation state refers to macrophages that are not M1 activated and comprise various activation states, induced by a wide array of different stimuli, leading to different macrophage classification systems by different authors. These stimuli include Th2 cytokines (such as IL-4 and IL-13), anti-inflammatory cytokines (such as IL-10 and TGF-β), hormones (such as glucocorticoids), and immune complexes. Consequently, non-M1 macrophages have very diverse functions, ranging from parasite control to immune suppression, wound repair, tissue remodeling, and angiogenesis. Features of these non-M1 macrophages are the low secretion levels of pro-inflammatory cytokines, high expression of macrophage mannose receptor (MMR) and scavenger receptor-A (SR-A), and an arginine metabolism shifted toward the production of ornithine and polyamines by arginase (35–39). Although the M1/M2 classification has proven useful, any form of classification underscores the complexity of the *in vivo* situation, in which numerous stimuli interact to define the final differentiated state and mixed functional profile of macrophages (40–42). In this context, new nomenclature and experimental guidelines for dealing with macrophage activation and polarization have very recently been proposed (43).

### M-CSF as driver of both differentiation and phenotypic polarization of macrophages

The myelopoietic growth factors macrophage-colony-stimulating factor (M-CSF, also known as CSF-1), granulocyte-macrophage-colony-stimulating factor (GM-CSF) and IL-34 are major cytokines in controlling the proliferation, differentiation, and functional regulation of monocytes, macrophages, and dendritic cells [reviewed in Ref. (44)]. M-CSF and IL-34 are produced by a variety of stromal and epithelial cell types and signal through the M-CSF receptor (M-CSFR, CSF-1R, or CD115), a type III receptor tyrosine kinase (45), encoded by the *Csf1r/c-fms* proto-oncogene (46, 47), that seems to be mainly restricted to cells of the mononuclear phagocyte lineage (48).

Especially, M-CSF instructs the myeloid fate in single hematopoietic stem cells, by inducing the myeloid master regulator transcription factor PU.1 (49). Embryonic yolk sac-derived precursors and fetal liver monocytes have been found to give rise to many tissue-resident macrophages that seed all tissues prenatally and are maintained via self-renewal throughout adult life (50). The importance of M-CSF for establishing and maintaining the tissue-resident macrophage pool is illustrated by the M-CSF-deficient osteopetrotic (*op/op*) mouse, which not only suffers from congenital osteopetrosis due to a severe deficiency of osteoclasts, but also features severe defects in many tissue-resident macrophage populations (51). Besides having effects on macrophage precursor differentiation, M-CSF is known to stimulate macrophage survival (52) and self-renewal during steady-state and inflammation (53). However, macrophage populations in distinct tissues are differentially affected by the M-CSF deficiency. For example, skin Langerhans cells and brain microglia were seemingly normal in *op/op* mice, but were largely absent from M-CSFR-deficient mice, a finding which has been explained by the trophic role of IL-34, whose production is restricted to keratinocytes and neurons under steady-state (54, 55).

In addition to a role in resident tissue macrophage differentiation and maintenance, M-CSFR signaling has also been assigned an important role in polarization of macrophage activation, flowing from the observation of significant differences in the transcriptomes of the macrophage populations primarily generated with the use of M-CSF or GM-CSF. M-CSF-driven macrophage differentiation leads to the expression of a substantial part of the M2 transcriptome, including expression of MMR and SR-A, while GM-CSF rather induces a pro-inflammatory M1-type of activation (49, 56–58). As such, blocking M-CSFR signaling in myometrial macrophages stimulated the occurrence of an M1-like MHC-II^high^ population at the expense of M2-like MHC-II^low^ macrophages in the pregnant mouse uterus (59). The same study also demonstrated an important role for M-CSF in mediating monocyte extravasation to the tissue, via M-CSF-dependent upregulation of the chemokine CCL2, adding further evidence to the notion that M-CSF affects macrophage dynamics at multiple levels.

Since high M-CSF levels are frequently found in tumor-bearing hosts, the M-CSFR signaling could also play a role in shaping the TAM pool and regulating their activation state.

## Association of M-CSF and M-CSFR Levels with Human Cancer Progression

### M-CSF, M-CSFR, and/or M-CSF response signature expression in tumor tissue

Various studies have documented analyses in which attempts were made to correlate clinical cancer patient parameters such as disease staging and survival with protein and/or mRNA expression levels of M-CSF, M-CSFR, and/or M-CSF response genes. The latter were thereby in turn considered to correlate with the presence of high levels of TAM and thus to represent a macrophage signature.

High M-CSF expression levels, as detected via IHC on tissue sections, have been reported to associate with higher histological tumor grading and in many cases also with more frequent metastases and poor prognosis in various cancer types, including breast cancer (60), serous and mucinous ovarian epithelial tumors (61), endometrioid carcinomas (62), and papillary renal cell carcinoma (63). In gynecological and non-gynecological leiomyosarcoma, expression of individual markers such as M-CSF was found to show at least a trend for correlation with poor outcome, but only the co-expression of M-CSF and three M-CSF-response genes (CTSL1, FCGR3a, and CD163) was independently associated with a worse survival in a multivariate analysis (64).

Studying the expression of M-CSFR via IHC in a large cohort of clinical breast cancer specimens using tissue microarrays revealed that M-CSFR expression was strongly associated with nodal status (65). In fact, in multivariate analysis, M-CSFR was not independent of nodal status as a predictor of survival. The study also revealed that M-CSFR expression was associated with decreased overall survival in non-metastatic breast cancer patients, but not in node-positive patients (65). Of note, in a recent manuscript, low levels of the M-CSFR gene were reported to predict worse overall survival based on online survival analysis tools allowing an evaluation of the prognostic value of genes in breast cancer patients using microarray data (66). In another recent study, a high number of tumor stromal cells – but not the cancer cells themselves – expressing M-CSFR was found to be an independent prognostic marker for lower event free survival and lower overall survival in classical Hodgkin lymphoma (67).

In line with variable results on association of M-CSFR expression with overall survival among different cancer types and patient groups, one report of a study using gene microarray and tissue microarray analyses for evaluating the prognostic value of an M-CSF response signature in breast cancer patients, mentioned a complex relationship of the signature with survival. Indeed, when patients were substratified in subsets, the M-CSF response signature was associated with poor prognosis among low-grade tumors and showed a trend for an association with improved prognosis among estrogen-receptor-negative tumors and among tumors with a TP53 mutation gene-expression signature (68). This variability in the association of M-CSF/macrophage signatures with clinical parameters points to the need to properly identify patient groups in which an M-CSF/macrophage signature correlates with worse prognosis and which are thus most likely to benefit from M-CSF/macrophage-targeted therapies.

It should also be remarked that the presence of an M-CSF/macrophage signature is not a uniform feature in all cancer patients. In fact, gene microarray and tissue microarray analyses revealed M-CSF and M-CSF response signature genes to be present in 17–25% of breast cancers (68) and in about 27% of myoinvasive endometrioid carcinomas (62). Yet, in the latter case, concordance between the expression of the M-CSF signature in primary endometrioid carcinomas and in their corresponding lymph node metastases was reported. Moreover, in case of breast carcinoma, expression of the M-CSF signature was not only detected in some patients in case of invasive ductal carcinoma, but was already detected at the stage of ductal carcinoma *in situ*. Also in that case, a correlation was found between the presence of the M-CSF signature in ductal carcinoma *in situ* and in invasive ductal carcinoma within the same patient (69). This conservation of the expression of the M-CSF signatures upon disease progression is promising when considering targeting of the M-CSF pathway as a therapeutic option for invasive and/or metastatic disease, and suggests that the presence of a M-CSF/macrophage signature in the primary tumor may be useful for patient stratification to identify those patients who are most likely to respond to M-CSF/macrophage-targeted therapies.

### Circulating M-CSF

In some cases, M-CSF is produced to such high levels in cancer patients that it can be detected systemically. Overall trends from studies in patients with newly diagnosed breast tumors indicate that circulating plasma M-CSF levels are not higher in patients with localized tumors than in controls, but are elevated in patients with regionally advanced disease and distant metastases (70, 71). Median M-CSF levels were also reported to be dramatically higher in patients with newly diagnosed tumors of the head and neck, in men with prostate cancer metastatic to bone and women with advanced metastatic breast cancer than those seen in patients with newly diagnosed breast tumors (70).

Prospective studies of the prognostic value of serum M-CSF levels have yielded conflicting results. One study on 471 women with pre-invasive and invasive breast carcinoma reported no significant association between pre-treatment plasma levels of M-CSF and overall/relapse free survival at a median follow up of 5.6 years. In this study, patients were classified into three groups based on the level of initial M-CSF, using median and twice median plasma values as cut-off points (70). In contrast, a recent study of 572 women with early breast cancer, that had not undergone local or systemic anti-cancer treatment prior to serum collection, revealed significantly poorer outcome at a median follow-up of 5.2 years in patients with above-median M-CSF concentrations as compared to those with below-median M-CSF concentrations. In this study population, log M-CSF serum concentrations at study enrollment were predictive of poor survival in both univariate analysis, as well as multivariate analysis adjusted for age, tumor size, nodal status, and tumor grade (71). In a retrospective, nested case–control study of breast cancer risk in 726 breast cancer patients and 734 matched controls with no cancer history, the association of circulating M-CSF levels with the risk of developing breast cancer was found to vary by menopausal status. High M-CSF levels were associated with a reduced risk of premenopausal breast cancer, whereas they were associated with an increased risk of postmenopausal breast cancer (72). Interestingly, in the aforementioned prospective study, the reported poorer outcome in patients with above-median M-CSF concentrations was confined to postmenopausal women, while no such effect was observed in premenopausal women with early breast cancer (71).

Overall, although the practical use of serum M-CSF levels as prognostic factor for cancer risk and/or outcome may be complicated by a high heterogeneity among patient groups and difficulties in determining optimal cut-off levels for plasma M-CSF, these results do suggest that, at least in some patient groups, M-CSF and M-CSF-dependent macrophages may be directly involved in tumor progression and malignant behavior and thus constitute interesting therapeutic targets.

## TAM Phenotypic and Subpopulation Heterogeneity

Originally, TAM were characterized as M2-like cells, proficient in inducing trophic functions like tumor angiogenesis, invasion, proliferation, and expressing the anti-inflammatory cytokine IL-10. These cells were also reported to express M2-specific markers like arginase-1, macrophage galactose-type C-type lectin-2 (Mgl2), found in inflammatory zone 1 (Fizz1), Ym1, TGF-β, SR-A, and MMR (73–76). However, some studies of chronic inflammation-induced cancer indicate the presence of TAM with an inflammatory M1-like phenotype, releasing inflammatory cytokines like IL-12, TNF, IL-6, and IL-1, or with overlapping M1 and M2 characteristics (77–79).

A dynamic switch in the phenotype of TAM during tumor progression might account for the mixed activation state of TAM subsets found in different established tumors. Indeed, in some models, tumor progression is associated with a switch from M1-like to M2-like TAM (80, 81). Hence, M2-like TAM can be linked to tumor promotion and their presence is indicative of poor prognosis (82, 83). Accordingly, a high M1/M2 TAM ratio has been associated with extended survival in many cancer types (84). Moreover, inhibition of monocyte differentiation to M2-like TAM through inhibition of NF-κB signaling, results in an M1-like phenotype and reduced tumor growth (85). Hence, a picture emerges whereby M2-like TAM are pro-tumoral, and M1-like TAM exert anti-tumoral activities.

Accumulating evidence suggests that different TAM activation states found within the same tumor may reflect responses to divergent local microenvionmental signals (86). As previously mentioned, tumors are complex organoid structures containing peritumoral stroma, perivascular regions, and hypoxic regions, which can all be populated by TAM, albeit with a different molecular profile and exerting specialized functions (86–89). Different studies, using state-of-the-art microscopy, clearly illustrated the existence of at least two distinct microenvironments in the same tumor, which were both infiltrated by TAM subsets. TAM residing in avascular regions are sessile, have a high phagocytic capacity, and express high levels of many prototypical M2 markers such as MMR. In contrast, perivascular TAM are migratory, are not able to ingest dextran, have a less pronounced M2-profile and produce epidermal growth factor (EGF), which attracts M-CSF-producing cancer cells, resulting in migration and intravasation of cancer cells (90–94). In line with these findings, differentially activated macrophages within the same tumors, residing in distinctively oxygenated tumor regions, could be discriminated based on the expression of MHC-II. MHC-II^high^ TAM are excluded from hypoxic avascular areas and more M1 oriented, while hypoxic MHC-II^low^ TAM express higher levels of M2-associated markers and are more angiogenic (25–27, 95). However, increasing the oxygenation of neoplastic lesions by vessel normalization in *PHD2*-haplodeficient mice was recently found not to alter the expression of the most prominent M2 markers, such as MMR, IL-4Rα, and Arginase-1. Rather, reduced hypoxia down-regulated a subset of genes and proteins involved in glycolysis, angiogenesis, and metastasis, thereby lowering their angiogenic functions, specifically and solely in the hypoxic MHC-II^low^ TAM subset (27). Hence, hypoxia is not the main driver of TAM differentiation, but M2-like TAM preferentially home to hypoxic areas where the pro-tumoral activities of these cells are promoted. The importance of the intratumoral TAM location in shaping the phenotype of TAM subpopulations was further confirmed by a study showing that Neuropilin-1 deficiency in macrophages prohibits their migration to hypoxic tumor areas, resulting in an increased inflammatory phenotype and the initiation of an anti-tumor immune response (96).

## Effects of M-CSFR Signaling on Numbers and Phenotype of TAM and Other TIM

**Figure 2:**
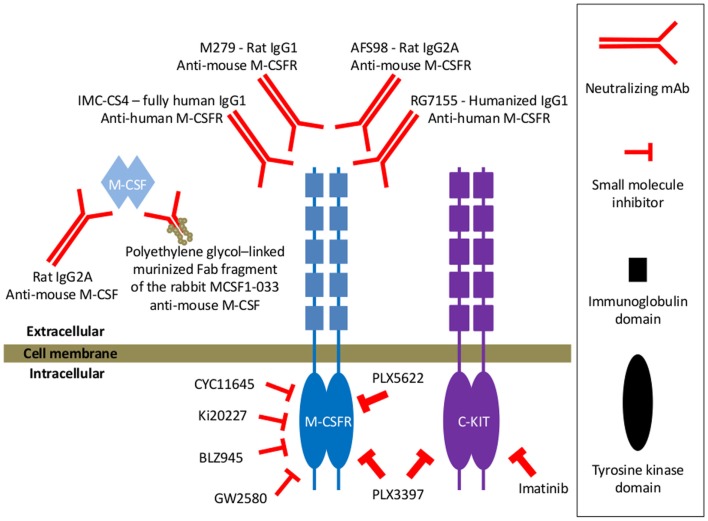
**Examples of various types of M-CSFR signaling blocking agents mentioned throughout this manuscript**. In some studies, neutralizing anti-mouse M-CSF mAb has been used for blocking M-CSF/M-CSFR signaling (66). One study also reported on the use of a murinized, polyethylene glycol-linked recombinant Fab fragment of the MCSF1-033 neutralizing rabbit anti-mouse M-CSF antibody (99). Yet, blocking mAbs targeting the extracellular domains of the M-CSFR have more frequently been documented for blocking the M-CSF/M-CSFR signaling axis. Typical examples of the latter that have been used in mouse tumor models are the rat IgG1 M279 (100) and the rat IgG2A AFS98 (101, 102). A recent report documented the generation of RG7155, a humanized anti-human M-CSFR IgG1 mAb that inhibits M-CSFR activation (106). And also the fully human IgG1 anti-human M-CSFR mAb IMC-CS4 is currently in clinical trials (107). M-CSFR signaling has also been inhibited via pharmacological, small molecule inhibitors targeting the intracellular catalytic domains of the receptor involved in signal transduction. A number of these tyrosine kinase inhibitors, such as CYC11645, Ki20227, GW2580, or BLZ945, have been screened for highly selective inhibition of M-CSFR signaling, very potent IC50 values for M-CSFR and at least a 100-fold lower inhibitory activity for other tested receptor tyrosine kinases (66, 108–110). Also the PLX3397 tyrosine kinase inhibitor has been used, which has higher M-CSFR inhibitory activity as compared to GW2580, but which is less specific since it inhibits the c-Kit receptor tyrosine kinase with similar potency as the M-CSFR tyrosine kinase (105). In one study, the actual contribution of M-CSFR blockade in the effect of PLX3397 has been assessed by comparing it with the specific cKit tyrosine kinase inhibitor imatinib and PLX5622, an M-CSFR-specific inhibitor of equal potency to PLX3397 that does not appreciably inhibit Kit (111).

The critical role of M-CSF in the turn-over of TAM is reflected in the drastic reduction in macrophages in the primary tumor at different stages of tumor progression to malignancy that has been observed in the absence of M-CSF in osteopetrotic *op/op* mice (97, 98). Conversely, restoration of M-CSF signaling via transgenic expression of M-CSF in the mammary epithelium led to enhanced numbers of macrophages in primary mammary tumors (97). Similarly, strong reductions in the number of TAM have been reported in various tumor models upon blocking of M-CSF/M-CSFR signaling to TAM using either blocking monoclonal antibodies (mAbs) targeting M-CSF (66, 99) or the extracellular domain of M-CSFR (100–102) or small molecule inhibitors of the M-CSFR tyrosine kinase activity in order to block the downstream signaling (102–105) (Figure 2).

Despite numerous reports of the differential effects of M-CSF versus GM-CSF on macrophage polarization (44, 57), only few studies directly addressing the effect of M-CSFR blockade on the M1/M2 activation state and/or subpopulation heterogeneity of TAM have recently been documented (Table 1).

**Table 1:** **Documented effects of M-CSFR inhibition on TAM abundance and activation state**.

Mouse tumor model	Tool used to inhibit M-CSFR signaling	Amount of TAM	TAM M1/M2 activation state	Effect/outcome	Reference
Colon carcinoma	*In vitro*: siRNA against M-CSFR + GM-CSF	Unaltered	Unaltered	Increased expression of STAT1, STAT5, STAT6 in TAM	(112)
Melanoma	M-CSFR inhibitor: PLX3397 + adoptive cell therapy	Reduced	Skewing from M2 MHC-II^low^ to M1 MHC-II^high^	Improved adoptive cell therapy: increased amount and activation of tumor-infiltrating lymphocytes; reduced tumor growth	(113)
Mammary carcinoma	M-CSFR inhibitor: GW2580	Reduced (only M2-like MHC-II^low^ TAM)	Not assessed	Role of M-CSFR in maintenance of M2-like TAM	(95)
Pancreatic ductal adenocarcinoma	M-CSFR inhibitor: PLX3397/neutralizing α-M-CSF MAb	Reduced (mainly M2-like MMR^high^ TAM)	Remaining TAM are less immunosuppressive, better Ag presenting M1	Increased anti-tumor T cell activity; enhanced response to immunotherapy	(114)
Glioblastoma	M-CSFR inhibitor: BLZ945	Unaltered	Repolarization from pro-tumoral M2 to phagocytic M1 TAM	Reduced tumor growth	(108)
Cervical and mammary carcinoma	M-CSFR inhibitor: BLZ945	Reduced (both MHC-II^low^ and MHC-II^high^ TAM)	Not assessed	Increased amount of CD8^+^ T cells; reduced tumor growth	(104)
Pancreatic ductal adenocarcinoma	M-CSFR inhibitor: PLX3397 or GW2580	Reduced (mainly M1-like immunosuppressive MHC-II^high^ TAM)	Remaining TAM are less immunosuppressive	Enhanced response to chemotherapy; increased CTL response; reduced metastases	(105)

One study in a mouse model based on subcutaneously inoculated colon carcinoma cells was aimed at evaluating whether cytokine signaling could induce reprograming of the TAM phenotype *in vitro*. The authors reported that GM-CSF treatment in conjunction with suppression of M-CSF signals using siRNA against the M-CSFR resulted in an altered signal transduction pathway of TAM, whereby expression of STAT1, STAT5, and STAT6 was increased. In this study, treatment of TAM with GM-CSF, alone, or in conjunction with suppression of M-CSFR signals, did not alter the TAM expression pattern of M1/M2 marker molecules (112).

In a study, whereby the tyrosine kinase inhibitor PLX3397 was used as a combination treatment with adoptive cell therapy of melanoma-targeted T cells in a syngeneic mouse model of BRAFV600E-driven melanoma, PLX3397 as single or combination treatment resulted in a dramatic reduction of TAM and a skewing of the subpopulation balance in the remaining TAM from predominant M2-oriented MHC-II^low^ to predominant M1-oriented MHC-II^high^ macrophages (113). A similar shift in the relative amount of TAM subpopulations was documented in the transgenic mouse MMTV-Neu model, in which mammary carcinogenesis is driven by the mammary epithelial restricted expression of the ErbB2/Neu oncogene. Blocking M-CSFR in this model by using the M-CSFR inhibitor GW2580, led to a significant reduction in the amount of M2-like F4/80^high^ TAM, which had moderate levels of MHC-II, and not in the MHC-II^high^ F4/80^low^ TAM, elucidating a role for M-CSFR signaling in the maintenance or expansion of the M2-like TAM subset (95). A recent study in a mouse model of pancreatic ductal adenocarcinoma confirmed this notion. In this model, treatment with PLX3397 or a neutralizing anti-M-CSF mAb resulted in a drastic reduction in TAM (114). Thereby, the authors demonstrated that blocking M-CSF/M-CSFR signaling resulted in preferential depletion of M2-like MMR^high^ TAM, whereas M1-like MMR^low^ TAM were much less affected. The observation that the MMR^high^ TAM subset had significantly higher M-CSFR expression levels as compared to the MMR^low^ TAM subset further supports the notion that these M2-like cells may be more dependent on the M-CSF signal. As a consequence, the gene-expression profile of TAM upon M-CSFR signaling blockade featured a reduced expression of M2 markers and an increased expression of M1 markers and MHC-II. In parallel, the TAM phenotype shifted from predominant immunosuppressive properties to improved antigen presentation capacity (114). In a mouse glioblastoma model, *in vivo* M-CSFR inhibition using the small molecule M-CSFR inhibitor BLZ945 was reported not to result in TAM depletion. Instead, glioma-secreted factors, including GM-CSF and IFN-γ, facilitated TAM survival in the context of M-CSFR inhibition and resulted in a repolarization from pro-tumoral M2 to a highly phagocytic M1 phenotype, with a decreased expression of M2 markers (108).

Despite the above examples indicating that M-CSFR blockade can shift the balance in TAM subpopulations from tumor-promoting M2-oriented MHC-II^low^ or MMR^high^ to anti-tumoral M1-oriented MHC-II^high^ or MMR^low^ macrophages, conflicting reports also exist. For example, the M-CSFR inhibitor BLZ945 was reported to result in a decrease in the level of TAM in cervical and breast carcinomas by attenuating their turn-over rate. Hereby, similar kinetics of depletion and recovery were observed for both MHC-II^low^ and MHC-II^high^ TAM subpopulations (104). And in mice bearing transplantable pancreatic ductal adenocarcinomas, the M-CSFR inhibitors GW2580 or PLX3397 were even reported to significantly deplete macrophages expressing high levels of MHC-II, but not the more M2-oriented MHC-II^low^ or Tie2^+^ TAM (105). Yet, in the latter case and in contrast to the examples above, the MHC-II^high^ TAM were found to constitute the predominant TAM subpopulation and to exert pro-tumoral activities by suppressing anti-tumoral CD8^+^ T cell responses (105). Therefore, also in that case, the observed reduction in the level of the predominant tumor-promoting TAM subpopulation, occurring upon M-CSFR blockade, resulted in attenuation of cancer malignancy (Table 1).

Further studies will be required to obtain better insights into the extent and the underlying mechanisms by which M-CSFR signaling and blockade thereof can contribute to shaping the phenotypic and subpopulation heterogeneity of TAM, thereby re-educating TAM toward anti-tumoral effector populations, thus contributing to combating disease progression. It will also be of importance to assess to what extent the remaining TAM populations detected after M-CSFR signaling blockade in various cancer types and tumor models are actually M-CSF-dependent macrophages for which the depletion was incomplete or the M-CSF dependence has been (partially) compensated for by other factors. Or do these remaining cells in some instances represent M-CSF-independent cells with a distinct lineage origin (such as for example certain dendritic cell types) for which the lineage surface markers and morphological analysis used in the current studies have not allowed to discriminate them from macrophages?

Of note, a number of recent publications evaluating the effect of M-CSFR inhibitors such as GW2580 or PLX3397 on various populations of tumor-infiltrating myeloid cells (TIM) have documented a reduction of not only mature CD11b^+^Ly6G^-^Ly6C^low^F4/80^high^ TAM, but also of CD11b^+^Ly6G^-^Ly6C^high^F4/80^mid^ cells, resembling the surface receptor phenotype and morphology of inflammatory (classical) monocytes or monocytic myeloid-derived suppressor cells (MO-MDSC) (105, 114–116). Taking into account the diversity of cell populations that can express these combinations of surface markers and the fact that an actual suppressive activity of the cells has not been demonstrated by the authors of most of these studies, we will term these cells MO-MDSC-like cells in the current review. It makes sense that, as monocyte-lineage cells, these tumor-infiltrating MO-MDSC-like cells are dependent on M-CSFR signaling to a similar extent as mature TAM and these MO-MDSC-like cells may in fact very well represent precursors of mature TAM (117). In contrast to MO-MDSC-like cells, documented effects of M-CSFR signaling inhibitors on CD11b^+^Ly6G^high^Ly6C^low^ cells, resembling the surface receptor phenotype and morphology of immature granulocytes/neutrophils or polymorphonuclear myeloid-derived suppressor cells (PMN-MDSC) and which we will term PMN-MDSC-like cells have been more variable. Most studies revealed no reduction (and sometimes even a limited increase) in the number of PMN-MDSC-like cells in response to PLX3397 or GW2580 treatment, for example in mice bearing murine pancreatic ductal adenocarcinoma (105, 114) or in the 3LL lung carcinoma model (115). In contrast, PLX3397 was found to reduce both MO-MDSC-like cells and PMN-MDSC-like cells in one study in the RM-1 and Myc-CaP prostate cancer models (116). The variable effect of M-CSFR blockade on PMN-MDSC-like cells suggests that this effect is most likely indirect and may depend on other (growth) factors in the tumor microenvironment that are affected indirectly via the M-CSFR blocking.

## Effects of M-CSFR Signaling Blockade on Cancer Progression and the Role of TAM Therein

Depending on the tumor type/model and the blocking agents used to impede M-CSFR signaling (Figure 2) (variable) effects of M-CSFR blockade on different aspects of cancer progression have been reported (Figure 1B).

### Effects on tumor incidence and primary tumor growth

To assess the role of M-CSF in tumor development and progression, *csf1^op^/^op^* mice have been crossed with transgenic mice in which mammary tumors develop due to mammary epithelial restricted expression of the Polyomavirus middle T oncogene (PyMT). In these experiments, the drastic reduction in TAM numbers in the absence of M-CSF was reported neither to affect the incidence nor the growth of the primary tumors but rather to delay their development to invasive, metastatic carcinomas (97). In fact, the PyMT model is characterized by the development of a single primary tumor focus on the ducts emanating from the nipple, after which other tumors arise in the ducts distant to the nipple. Although the development of multiple foci on the distal ducts was reduced in the *csf1^op^/^op^* PyMT mammary glands, the growth rate of the primary tumor size and the proliferation rate of the cancer cells were comparable to those in M-CSF sufficient mice.

Similarly, treatment of AE5MG mesothelioma or LLC lung carcinoma bearing mice with the M-CSFR blocking mAb M279 was described not to result in a significant effect on tumor growth or final tumor burden, despite a strong reduction in the number of TAM (100). In contrast, publications reporting on the use of another mAb, AFS98, for M-CSFR blockade and ensuing TAM inhibition, documented inhibition of primary tumor growth in different mouse tumor models including the implanted AX osteosarcoma model (102) and later also in the EL4 transplanted lymphoma model, the PyMT transgenic breast carcinoma model and the MDA-MB231 breast cancer metastasis-induced osteolysis model (101). It has been suggested that the effect of the rat IgG1 M279 may represent the biological response to blocking CSF-1R signaling *per se*, whereas the isotype of the rat IgG2A AFS98 may result in additional effector functions such as direct macrophage depletion upon recognition by and/or aggregation with other macrophages through binding of the IgG2A antibody to the high affinity IgG receptor CD64 on mouse macrophages (52).

In human, MCF-7 mammary carcinoma cell xenografts in immunodeficient mice, M-CSF blockade by antisense oligonucleotides and small interfering RNAs has been shown to reduce host macrophage infiltration and suppress tumor growth (118). Concerning the effect of pharmacologic M-CSFR blockade on primary tumor growth, the M-CSFR tyrosine kinase inhibitor Ki20227 was described to reduce TAM content of tumors and retard tumor cell proliferation in osteosarcoma (102) and similar results were reported for GW2580 in papillary thyroid cancer (103). Yet, the reduction in intratumoral proliferation in GW2580-treated papillary thyroid cancers was most evident within the stromal compartment (103). These results suggest that the observed effects of M-CSF blockade may in that case at least partially reflect inhibition of stromal cells such as TAM rather than cancer cell proliferation *per se*. In murine prostate cancer models, the M-CSFR inhibitors GW2580 or PLX3397 as a single treatment were reported to have little effect on tumor growth compared with the control group, despite effective TAM ablation.

A recent study clearly illustrates that the specificity of the applied inhibitors for M-CSF as compared to other tyrosine kinases and the relative contribution of the effect on TAM as compared to direct effects on the cancer cells should be carefully considered when interpreting the effect of M-CSFR blockers on tumor growth. In this study, PLX3397 was found to result in effective reduction of tumor weight and cellularity in both the Kit^V558del/+^ transgenic murine gastrointestinal stromal tumor (GAST) model and in human GAST xenografts (111). These GAST cells are known to be strongly dependent on signaling via the oncogene cKit for their survival and the growth inhibitory effect of PLX3397 was even stronger than that of the cKit tyrosine kinase inhibitor imatinib, correlating with a superior capacity of PLX3397 as compared to imatinib to decrease the viability of two human GAST cell lines *in vitro*. On the other hand, TAM were deleted to a much greater degree in mice treated with PLX3397 than with imatinib, correlating with a more potent M-CSFR inhibition by PLX3397 as compared to imatinib. Therefore, one could hypothesize that the superior effect of PLX3397 on tumor growth inhibition could at least in part be related to superior inhibition of M-CSF signaling and consequent TAM attenuation, acting synergistically to the Kit inhibition. To address this possibility, the authors combined imatinib with PLX5622, an M-CSFR-specific inhibitor of equal potency to PLX3397 that does not appreciably inhibit Kit. Despite comparable levels of TAM reduction as PLX3397 therapy, treatment with PLX5622 did not enhance the effect of imatinib on tumor weight, cell number, or histology, suggesting that inhibition of cKit signaling but not M-CSFR signaling is the main factor determining the capacity of tyrosine kinase inhibitors for GAST growth inhibition (111).

Overall, despite consistent reduction in TAM content in primary tumors in the various tumor models discussed above, the effects of M-CSF or M-CSFR blockade and consequent TAM attenuation on primary tumor growth seem to be quite variable, depending on the tumor model and the blocking agents used, and thus do not seem to correlate with TAM depletion *per se*.

### Effects on tumor angiogenesis

Crossing PyMT and *csf1^op^/^op^* mice revealed that a low density of macrophages in the primary tumors correlated with a delay in the angiogenic switch, identified as the formation of a high-density vessel network. Genetic restoration of macrophage numbers in the tumors of these mice by the transgenic expression of M-CSF specifically in the mammary epithelium thereby rescued the vessel phenotype (119). Similarly, crossing *csf1^op^/^op^* mice to the RIP1-Tag2 (RT2) mouse model of pancreatic islet cancer was documented to decrease TAM by approximately 50% during all stages of RT2 tumor progression and to generate a substantial reduction in cumulative tumor burden, which resulted from a significant decrease in angiogenic switching and the number of tumors, rather than an evident effect on the growth of established tumors or on the cancer cell proliferative capacity (98).

In a mammary tumor model based on xenografts of human MCF-7 breast cancer cells in athymic nude mice, mouse (host) M-CSF expression was found to be induced as the tumors progressed. In these mice, treatment with a murinized, polyethylene glycol-linked recombinant Fab fragment of the MCSF1-033 neutralizing rabbit anti-mouse M-CSF antibody reduced the density of both macrophages and proliferating endothelial cells, the latter reflecting decreased levels of angiogenic activity in the mammary tumor xenografts (99). In an immunocompetent mouse model of osteosarcoma, in which mice were subcutaneously transplanted with the mouse AX osteosarcoma cell line, the M-CSF inhibitor Ki20227 or the AFS98 rat anti-murine M-CSFR mAb dramatically decreased peritumoral and perivascular TAM, suppressed tumor angiogenesis and lymphangiogenesis, disorganized extracellular matrices and concomitantly dramatically suppressed metastasis and improved prognosis (102). In contrast to VEGF blockade, interruption of M-CSF signaling did not promote rapid vascular regrowth. In addition, continuous M-CSF inhibition did not affect healthy vascular and lymphatic systems outside tumors (102). The notion that M-CSFR^+^ TIM, including both TAM and MO-MDSC-like cells, contribute significantly to tumor angiogenesis, was supported by Priceman et al. (115), showing that depletion of M-CSF-dependent TAM and MO-MDSC-like cells in the 3LL lung carcinoma model, using either the M-CSFR inhibitor GW2580 or a transgenic approach in chimeric mice, resulted in significant reduction in angiogenesis in TIM-ablated tumors (without a concomitant decrease in tumor growth). The authors confirmed that, also in the orthotopic RM-1 prostate tumor model, M-CSF blockade resulted in reduced levels of TAM, and MO-MDSC-like cells, associated with reduced angiogenesis and, to a lesser extent, lymphangiogenesis, as reflected by vessel density in these tumors.

In the 3LL lung carcinoma model, GW2580 was in addition shown to attenuate tumor evasion of anti-angiogenic therapy. In combination with DC101, a specific blocking antibody against VEGFR-2, GW2580 resulted in greater inhibition of tumor angiogenesis along with synergistic tumor growth reduction compared with anti-angiogenic therapy alone. In search for a hypothesis on the mechanism underlying the reversal of anti-angiogenesis in the combination therapy, the authors provided histological data revealing more abundant MMP9 expressing cells with heterogeneous myeloid cell morphology in viable areas of DC101-treated tumors, which were reduced in the combination group (115).

At a mechanistic level, M-CSF was also shown to induce VEGF production in human monocytes through the MAPK/ERK pathway via Sp1 and was reported to enhance angiogenesis *in vivo*, as evidenced in an angiogenesis assay using an *in vivo* polymerized Matrigel^TM^ plug in mice (120). Recently, the mechanistic basis of the tumor angiogenesis-promoting effect of M-CSF was further expanded by showing that M-CSF augments differentiation of the subpopulation of M2 macrophages expressing the endothelial cell tyrosine kinase receptor, Tie2. Hereby, M-CSF-mediated upregulation of Tie2 on these Tie2-expressing monocytes/macrophages (TEM) increased branching of human umbilical vein endothelial cells (HUVECs) *in vitro* and enhanced angiogenesis in PyMT tumor-bearing mice. This M-CSF-stimulated Tie2 receptor expression was found to be dependent on a synergistic contribution from the PI3 kinase and HIF-1α pathways. (121).

As a final remark, it should be realized that high levels of angiogenesis, driven by M2-like TAM, often lead to dysfunctional blood vessels in tumors, resulting in more malignant cancer cells under the influence of tumor hypoxia and an easy access of these cells to the blood circulation. TAM depletion or the conversion of M2-like TAM to M1-like TAM, thereby results in vessel normalization and reduced metastasis (122). In addition, normalized vessels allow a more efficient administration of therapeutic agents to the tumor microenvironment.

### Effects on cancer cell invasion and metastasis

Accumulating evidence in the first decade of this century has supported the tenet that delayed development of invasive, metastatic carcinomas in PyMT *csf1^op^/^op^* mice is reflective of a role for M-CSF in promoting cancer cell invasion by regulating the infiltration and function of TAM. Indeed, at the PyMT tumor site, expression of M-CSFR was reported to be restricted to macrophages. Moreover, restoration of macrophage infiltration upon transgenic expression of M-CSF in the mammary epithelium restored progression of primary tumors to the stages of invasive carcinoma (97). In fact, macrophages and tumor cells in mammary tumors were documented to be comigratory and to be mutually dependent for invasion and for cancer cell intravasation (90, 123). Hereby, M-CSF produced by carcinoma cells promotes the expression of EGF by macrophages, which in turn promotes the formation of elongated protrusions and cell invasion by carcinoma cells. In addition, EGF promotes the expression of M-CSF by carcinoma cells, thereby generating a positive feedback loop. Disruption of this paracrine amplification loop by blockade of either EGF receptor or M-CSFR signaling was found to be sufficient for inhibiting both macrophage and tumor cell migration and invasion (91).

A similar EGF/M-CSF paracrine interaction with macrophages, resulting in enhanced cancer cell invasion as reported for murine carcinoma cells, was confirmed in a mouse xenograft model of human breast tumor derived cancer cells. Yet, for these human breast carcinoma cells, the EGF/M-CSF paracrine feedback loop was found to be complemented by autocrine M-CSF signaling in the cancer cells (124). These data correlated with the expression of M-CSFR by human but not mouse breast carcinoma cells. The possibility of macrophage-independent effects of M-CSF on human cancer cell invasion is also supported by a direct stimulation of *in vitro* invasive capacity, but not proliferation, of human adenocarcinoma cell lines by recombinant human M-CSF (125).

The studies in *csf1^op^/^op^* mice also indicated a role of M-CSF in enhancement of metastatic growth of cancer cells. In particular, M-CSF was shown to be required for the recruitment of a population of CD11b^+^F4/80^+^Gr1^+^ host macrophages to extravasating pulmonary metastatic cells in the PyMT model. This recruited CD11b^+^F4/80^+^Gr1^+^ macrophage population displayed a distinct phenotype as compared to CD11b^−^, CD11c^+^ lung resident macrophages and also did not express Tie2, rendering them distinct from the M-CSF-induced pro-angiogenic Tie2-expressing monocytes/macrophages. The recruited macrophages enhanced cancer cell metastasis through effects on cancer cell metastatic seeding, extravasation, survival, and subsequent growth (126). The authors confirmed that the reduced metastasis in *csf1^op^/^op^* PyMT mice could be recapitulated in wild-type PyMT mice via macrophage ablation using clodronate-containing liposomes. Importantly, even after metastatic growth had been established, macrophage ablation using clodronate-containing liposomes inhibited subsequent metastatic growth (126). This effect also seems to be M-CSF-specific since transgenic expression of M-CSF in the mammary epithelium of both *csf1^op/op^* and wild-type tumor-prone mice led to an acceleration to the late stages of carcinoma and to a significant increase in pulmonary metastasis. The clinical significance of these findings is illustrated by the observation that the density of close tripartite interactions between cancer cells, macrophages, and endothelial cells (tumor microenvironment of metastasis or TMEM) is predictive of metastasis formation in breast cancer patients (127).

Since M-CSF signaling not only plays a critical role in the turnover of TAM, but is also crucial for osteoclasts, blocking M-CSFR signaling may not only attenuate metastasis via effects on TAM, but may have additional beneficial effects on metastatic disease via inhibitory effects on osteoclasts. As an example of this, the AFS98 M-CSFR blocking mAb was recently documented to potently block the differentiation of osteoclasts and their bone destruction activity in a breast cancer model of bone metastasis (101).

A recent study placed a cautionary note on blocking M-CSFR signaling as a therapeutic modality in cancer. In that study, mice bearing two independently derived mammary cancer cell lines (4T1.2 and EMT6.5) injected orthotopically into the mammary gland, were treated with the AFS98 neutralizing anti-M-CSFR mAb, with a neutralizing anti-mouse M-CSF mAb, or with two different small molecule inhibitors of M-CSFR (GW2580 or CYC11645). The authors observed variable effects on reduction of TAM in the primary tumors or metastatic lung tissue, whereby TAM could be reduced using GW2580 or high dose of AFS98, but were not reduced when using lower dose of AFS98. Yet in all these cases, not only did these various modalities for blocking M-CSFR not reduce primary tumor growth, but the intended treatment actually increased metastasis to the lung and spine (66). The authors found that the increased spontaneous metastasis upon blocking of M-CSFR or M-CSF was associated with increased levels of serum granulocyte-colony-stimulating factor (G-CSF), increased numbers of neutrophils and Ly6C^high^ monocytes in the peripheral blood and increased frequency of neutrophils in the primary tumor and in the lung. It is currently unclear why M-CSFR blockade resulted in increased G-CSF levels in this model, but the authors did observe that blood neutrophil numbers were proportional to the metastatic capacity of the different mammary carcinomas evaluated, suggesting that certain carcinomas may be more prone to mobilize neutrophils, and leading to increased metastasis. Interestingly, combining blockade of M-CSFR signaling with a neutralizing antibody against the G-CSF receptor (G-CSFR), which regulates neutrophil development and function, reduced the enhanced metastasis, and neutrophil numbers that resulted from M-CSFR blockade. In fact, the combined blocking of M-CSFR and G-CSFR resulted in significantly reduced metastasis as compared to the control condition (66).

### Potentiation of radio-, chemo-, and immunotherapy

Whereas the M-CSFR inhibitors GW2580 or PLX3397 on their own were reported to have little effect on tumor growth in murine prostate cancer models, when added to radiotherapy, the M-CSFR inhibitors suppressed tumor growth more effectively than radiation alone (116). The synergistic effect of M-CSF blockade on the efficacy of radiotherapy was explained by the observation that irradiation resulted in increased M-CSF levels due to recruitment of the DNA damage-induced kinase ABL1 into cell nuclei where it bound the *csf1* gene promoter and enhanced *csf1* gene transcription. Consequently, enhanced recruitment of TIM, including TAM and MO-MDSC-like and PMN-MDSC-like cells, was detected and this enhanced TIM recruitment was counteracted via the M-CSFR inhibitors (116). These results suggest that blockade of the M-CSF/M-CSFR axis can be a promising approach for developing more effective combination cancer therapies. The authors supported the human relevance of these findings by reporting that also in prostate cancer patients, serum levels of M-CSF were increased after radiotherapy.

Such potential synergistic effects in combination therapy are not only restricted to radiotherapy, but also extend to chemotherapy. Indeed, combination therapy with a murinized, polyethylene glycol-linked antigen-binding fragment against mouse (host) M-CSF reportedly reversed chemoresistance in athymic nude, immunodeficient mice bearing human, and chemoresistant MCF-7 breast cancer xenografts (99). Also treatment with the AFS98 anti-M-CSFR monoclonal antibody in mice already bearing established PyMT tumors was reported to prolong their survival and potentiate the effect of chemotherapy with Paclitaxel (101). Finally, GW2580 or PLX3397 were found to improve chemotherapeutic efficacy in mice bearing murine pancreatic ductal adenocarcinoma cell lines. In this tumor model, gemcitabine chemotherapy was documented to increase M-CSF levels and consequently enhance the tumor infiltration of T-cell suppressive TAM (and MO-MDSC-like cells). This effect was blunted when chemotherapy was combined with M-CSFR blockade, resulting in increased anti-tumor CD8^+^ T-cell responses and improved inhibition of tumor growth and metastasis as compared to chemotherapy as monotherapy. Accordingly, the higher therapeutic efficacy of combined treatment with GW2580 plus gemcitabine compared with the effects of gemcitabine alone was shown not to occur upon depletion of CD8^+^ T lymphocytes (105).

Considering the above, it comes as no surprise that, in the same mouse model of pancreatic ductal adenocarcinoma, M-CSFR signaling blockade using PLX3397 or GW2580 was shown to enhance the therapeutic efficacy of so-called T-cell checkpoint immunotherapy using PD1 and CTLA4 antagonists in combination with gemcitabine (114). In this case, M-CSFR signaling blockade was reported to result in preferential depletion of MMR^high^ M2-like TAM and reprograming of the phenoptype of the remaining TAM, with alleviated immunosuppressive activities and enhanced antigen presentation capacity and which in turn correlated with enhanced CD4^+^ and CD8^+^ T cell responses. Here also, the increased therapeutic efficacy of the combination treatment was shown to be blunted upon depletion of CD4^+^ and CD8^+^ T cells (114). As another example of a synergistic effect of M-CSFR blockade on immunotherapy, PLX3397 has been reported to improve the efficacy of adoptive cell therapy of melanoma-targeted T cells in a syngeneic mouse model of BRAFV600E-driven melanoma. Mice receiving the combined treatment produced superior anti-tumor responses and exhibited improved overall survival compared with single treatments, correlating with a dramatic reduction of TAM (but in this setting no significant change in already low numbers of MO-MDSC-like or PMN-MDSC-like cells), a skewing of the subpopulation balance in the remaining TAM from predominant M2-oriented MHC-II^low^ to predominant M1-oriented MHC-II^high^ macrophages and an increase in tumor-infiltrating lymphocytes and T cells. The authors conclude that macrophages are the targets of PLX3397 by confirming that PLX3397 and macrophage-depleting clodronate-containing liposomes have the same effect on tumor growth and that this effect is not further increasing when combining both depletion methods (113).

## Concluding Remarks and Clinical Perspectives

Macrophage-colony-stimulating factor receptor inhibitors are currently in clinical development as cancer therapeutics. Plexxicon has, for example, initiated several clinical trials of the cKit and M-CSFR inhibitor PLX3397, either as a stand-alone cancer treatment (128–130) or in an adjuvant setting with chemo- and/or radiotherapy (131–134). Phase I clinical trials of anti-M-CSFR mAbs in patients with advanced solid tumors are currently being conducted by Eli Lilly and Company for the fully human IgG1 IMC-CS4 (107) and by Roche for the humanized IgG1 RG7155 (135). For the latter, it was mentioned in a recent publication that, based on preliminary results of an ongoing clinical trial, administration of RG7155 to diffuse-type giant cell tumor patients led to significant reductions of M-CSFR^+^CD163^+^ macrophages in tumor tissues, which correlated with at least partial clinical objective responses (106). The ultimate value of these M-CSFR targeted therapies will need to be assessed in follow-up studies aimed at demonstrating effects that go beyond reduction in the primary tumor burden, but extend to attenuation of metastasis and prolongation of patient survival.

In this context, it is encouraging that numerous studies in preclinical tumor models have revealed that blocking M-CSFR signaling, despite variable effects on primary tumor growth *per se*, has the potential to attenuate tumor-promoting effects of TAM on tumor angiogenesis and cancer cell invasion and metastasis. And especially, synergistic effects of M-CSFR blocking agents in diminishing TAM-dependent resistance to anti-angiogenic therapy, radiotherapy, chemotherapy, or immunotherapy offer promising perspectives for effective combination therapy. Recent studies thereby suggest that intratumoral M-CSF levels and their balance with GM-CSF levels are not only critical for TAM differentiation and maintenance, but can also contribute to shaping the M1/M2 phenotypic and subpopulation heterogeneity of TAM. Hence, M-CSFR blocking agents may not only have the potential to counteract cancer progression by reducing TAM content in tumors and metastatic lesions, but also by re-educating TAM from tumor-promoting toward anti-tumoral effector populations.

Recently, more attention is in addition being given to better characterize other tumor-infiltrating myeloid cell populations such as MDSC-like cells that are also affected by M-CSFR blockade and to evaluate whether these contribute to the observed effects of M-CSFR blockade on various aspects of cancer progression. Additional effects on other cells are not necessarily a disadvantage in the context of anti-cancer therapeutic activity, as exemplified in reported attenuation of metastatic disease via dual inhibitory effects on TAM and osteoclasts (101). Yet, the data recently reported by Swierczak and colleagues on neutrophil-dependent enhanced metastasis upon M-CSFR blockade (66) indicate that blocking M-CSFR signaling may have variable effects according to the tumor model and may in some cases exhibit unwanted side effects. These cautionary findings are testaments to the notion that successful clinical translation will be critically dependent on proper patient stratification to focus on those patient groups in which high M-CSF or M-CSFR expression is linked to disease pathophysiology and correlates with worse prognosis and in which M-CSFR/macrophage-targeted therapies are thus most likely to exert a beneficial effect.

## Author Contributions

All authors have made substantial contributions to the conception of the manuscript. Geert Raes and Damya Laoui have drafted the initial version of the manuscript. All other authors have critically reviewed the manuscript for important intellectual content. All authors approve of the version of the manuscript and agree to be accountable for all aspects of the work in ensuring that questions related to the accuracy or integrity of any part of the work are appropriately investigated and resolved.

## Conflict of Interest Statement

The authors declare that the research was conducted in the absence of any commercial or financial relationships that could be construed as a potential conflict of interest.

## Abbreviations

DAMP, damage-associated molecular pattern; EGF, epidermal growth factor; Fizz1, found in inflammatory zone 1; GAST, gastrointestinal stromal tumor; G-CSF, granulocyte-colony-stimulating factor; GM-CSF, granulocyte-macrophage colony-stimulating factor; HUVEC, human umbilical vein endothelial cell; mAb, monoclonal antibody; M-CSF, macrophage-colony-stimulating factor; M-CSFR, M-CSF receptor; Mgl2, macrophage galactose-type C-type lectin-2; MMP9, matrix metalloproteinase 9; MMR, macrophage mannose receptor; MO-MDSC, monocytic myeloid-derived suppressor cells; PAMP, pathogen-associated molecular pattern; PMN-MDSC, polymorphonuclear myeloid-derived suppressor cells; PyMT, polyomavirus middle T oncogene; RNI, reactive nitrogen intermediates; ROS, reactive oxygen species; RT2, RIP1-Tag2; SR-A, scavenger receptor-A; TAM, tumor-associated macrophages; TEM, Tie2-expressing monocytes/macrophages; TGF-β, transforming growth factor-β; TIL, tumor-infiltrating lymphocytes; TIM, tumor-infiltrating myeloid cells; TMEM, tumor microenvironment of metastasis; VEGF, vascular endothelial growth factor.
